# GeneBrowser 2: an application to explore and identify common biological traits in a set of genes

**DOI:** 10.1186/1471-2105-11-389

**Published:** 2010-07-21

**Authors:** Joel P Arrais, João Fernandes, João Pereira, José Luís Oliveira

**Affiliations:** 1 Department of Electronics, Telecommunications and Informatics (DETI), Institute of Electronics and Telematics Engineering of Aveiro (IEETA), University of Aveiro, 3810-193 Aveiro, Portugal

## Abstract

**Background:**

The development of high-throughput laboratory techniques created a demand for computer-assisted result analysis tools. Many of these techniques return lists of genes whose interpretation requires finding relevant biological roles for the problem at hand. The required information is typically available in public databases, and usually, this information must be manually retrieved to complement the analysis. This process is a very time-consuming task that should be automated as much as possible.

**Results:**

GeneBrowser is a web-based tool that, for a given list of genes, combines data from several public databases with visualisation and analysis methods to help identify the most relevant and common biological characteristics. The functionalities provided include the following: a central point with the most relevant biological information for each inserted gene; a list of the most related papers in PubMed and gene expression studies in ArrayExpress; and an extended approach to functional analysis applied to Gene Ontology, homologies, gene chromosomal localisation and pathways.

**Conclusions:**

GeneBrowser provides a unique entry point to several visualisation and analysis methods, providing fast and easy analysis of a set of genes. GeneBrowser fills the gap between Web portals that analyse one gene at a time and functional analysis tools that are limited in scope and usually desktop-based.

## Background

Biological systems pose very interesting challenges to scientists and despite the availability of relatively large sets of data, their inherent complexity stands out as the major hindrance to scientific progress in this field of work [1].

Comprehension of gene-to-phenotype interactions plays a key role in this process. Genes, the basic blocks of heredity, do not operate alone but instead in complex networks of interactions where multiple genes can directly or indirectly influence a single phenotype. Such networks exist even in the simplest of organisms, a clear indicator of their relevance [2].

Bearing this need in mind, the development of large-scale genome experiments (such as DNA microarrays) created the opportunity to gain a holistic view of biological systems, thus providing a better view of the full complexity of the systems in hand. Nevertheless, interpreting the results of DNA microarrays is still a daunting task that must be performed if any knowledge is to be extracted.

The classical approach to this analysis is a two-step procedure [3]: first, a subgroup of genes considered differentially expressed are selected by one or more bioinformatics tools; and second, the resulting set of genes is further explored to extract commonalities and biological meanings that may help to explain why these genes were co-expressed in the experiment. The primary focus of this paper is related to this second goal.

A common approach consists of the assignment of genes to functional biological categories based on the assumption that genes with similar expression profiles tend to have similar biological roles. While several bioinformatics tools have been proposed to perform this task, most of them use Gene Ontology [4] and pathway databases as the main sources of data. One such example is Onto-Express [5], a tool that uses Gene Ontology data to provide a functional profile for the condition studied. Onto-Express is a desktop application and was the first to compute significance values in the context of gene expression studies. Other examples of tools that use Gene Ontology are GOMiner [6] and GOstat [7]. A complete review of similar tools is available in [8]. There are also tools focused on other domains, such as the Pathway Explorer [9] for analysis of regulatory, metabolic and cellular pathways or Quext [10] for analysis of the literature. Some wide-scope tools have also been proposed. For example, FatiGO [11,12] is a web-based tool that, in addition to Gene Ontology, also uses other concepts, such as chromosomal location and pathways, to provide a comprehensive analysis of the results. DAVID [13] also allows the functional analysis of a set of genes based on the analysis of Gene Ontology, pathways, protein domains and literature. Despite the value of the presented tools, some limitations were found including the number of covered species (11 on FatiGO, 13 on GoMiner and 21 on Pathway Explorer), the time taken to process the input query and the possibility to explore the dataset with references to external data sources.

In this paper, we present GeneBrowser, a web-based tool that addresses the issue of extracting biological knowledge from a list of genes. GeneBrowser combines data from several sources and different visualisation methods to improve biological interpretation and knowledge extraction from a group of genes. GeneBrowser combines the advantages of web portals, such as Entrez gene [14] and GeneCards [15], with the advantages of the previously presented tools for functional analysis.

While a previous version of GeneBrowser was already presented in [16], the platform was completely revamped to include many new features, such as an improved methodology to calculate the significance of results, a completely redesigned interface with Web 2.0 principles, dataset filtering capabilities, improved overall performance and additional functionalities including gene locus, gene expression studies and homologies, among others. GeneBrowser 2.0 is freely available at http://bioinformatics.ua.pt/genebrowser2/.

## Implementation

**Figure 1:**
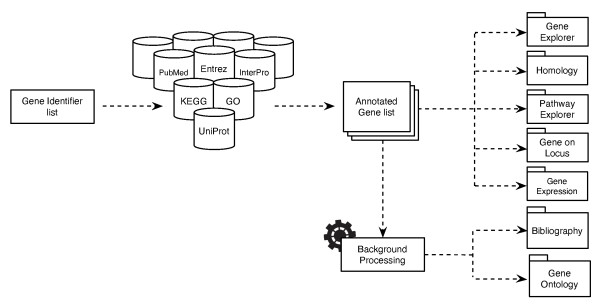
**Annotation and analysis workflow**. The schematic representation of the workflow implemented to annotate and analyse the dataset is shown. For a list of inserted gene identifiers, GeneBrowser accesses several biological databases to annotate each gene with the data from several public databases. The bibliography and Gene Ontology are executed in the background.

**Figure 2:**
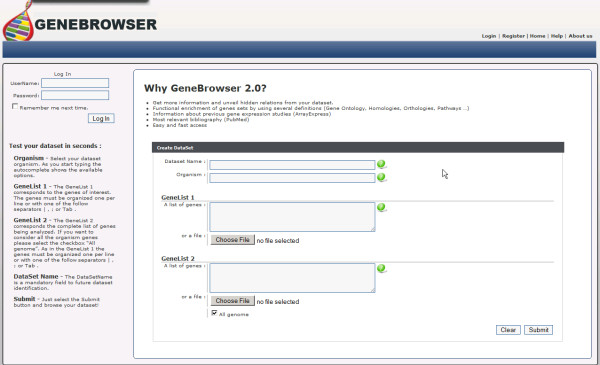
**GeneBrowser user-interface: main window**. The main interface allows the creation of a new dataset and navigation of the previously created datasets. To create a new dataset, it is necessary to specify the dataset name, the species of the organism under study, the list of genes identified as differently expressed and a second list with the complete list of genes under study.

GeneBrowser is a web application that combines data from several biological data sources and visualisation methods to explore a list of genes. Figure 1 illustrates the implemented workflow. As input, this tool takes a list of gene identifiers that are subsequently used to retrieve information from several public data sources, such as UniProt [17], Entrez gene [14], Gene Ontology, KEGG [18] and PubMed [19]. These data are then processed and merged, allowing the user to further explore the results via several visualisation perspectives and methods. Moreover, the system provides direct links to the original repositories, where complementary information is available. The main requirements of GeneBrowser development were to fill the gap between functional analysis tools and Web portals and to allow a fast response to user requests by means of state-of-the-art Web technologies. Figure 2 presents the GeneBrowser main interface. GeneBrowser is implemented in ASP.NET and uses Web 2.0 technologies, including AJAX, JSON and SOAP/REST Web Services. To assure efficiency and scalability, the data are stored in a SQL Server 2008 database cluster.

Although GeneBrowser can be used to answer many different biological questions, a particular question set was used to tune its development:

- What public databases provide relevant information about my dataset and how can I navigate through them?

- What biological processes are enriched with respect to my input list of genes?

- What are the most relevant metabolic pathways that contain my genes?

- What are the genomic regions of these genes?

- Which are the most relevant homologue classes in my list of genes?

- What gene expression experiments have been previously conducted with the same genes?

- What are the most relevant publications associated with my study?

### Integrated access to biological data

The functionalities provided by GeneBrowser require intensive access to several biological databases. For each set of genes, GeneBrowser must independently access an array of databases as a means to validate every single entry and obtain additional biological data to provide as much relevant information as possible. The nature of this procedure determines that its response time is directly proportional to the number of genes evaluated. Notwithstanding, the platform must have a low response time if it is to be of any practical use. Such a dichotomy could only have been solved through use of an integration platform that quickly validated the entries and returned the required data. For that purpose, we have developed GeNS [20], a database that works as a name server for biological entities.

GeNS has a generic database schema that supports an unlimited number of biological databases. Addition of a new database requires identification of the most suitable method to obtain data and development of a specific loader responsible for converting data to a format compatible with the schema. Currently, we integrate data for roughly 1000 species, representing over 7 million gene products with 70 million alternative gene/protein identifiers and 140 million associations to biological entities. For instance, the species *Saccharomyces cerevisiae *has 7421 gene products that can be mapped to 105.000 synonyms and 213.000 associations with biological entities, such as pathways, Gene Ontology terms or homologues. Detailed information regarding the schema and the integrated databases is available in [20].

Despite the variety of data stored in GeNS, it is more focused on the mapping of biological identifiers than on the actual data (e.g.: functional descriptions, structural and sequence data). Given the need to complement this lack of relevant information, GeneBrowser performs direct, run-time access to a selected set of data sources. Some examples include the following: a) extended protein details, obtained in XML format from the UniProt REST interface; b) bibliographical abstracts, obtained from PubMed; and c) other data necessary for construction of the Gene Explorer perspective such as the sequence obtained from GenBank and the protein structure obtained from PDB. Table 1 contains a complete list of the databases that are integrated and also the methods that are used to access them. For a list of one hundred human genes, validating and obtaining all of the associated concepts are performed in less than one second, which shows the concern taken during development to meet the real-time execution goal.

**Table 1:** List of integrated databases and methods applied to extract data

Type	Name	GeNS	Real-time data retrieval	URL
Gene	Ensembl	X		http://www.ensembl.org/
	
	HGNC	X		http://www.genenames.org/
	
	UniGene		X	http://www.ncbi.nlm.nih.gov/unigene/
	
	Entrez Gene	X	X	http://www.ncbi.nlm.nih.gov/gene/
	
	RefSeq	X		http://www.ncbi.nlm.nih.gov/refseq/
	
	KEGG Genes	X	X	http://www.kegg.jp/

Protein	UniProtKB	X	X	http://www.uniprot.org/
	
	Gene3D	X		http://gene3d.biochem.ucl.ac.uk/Gene3D/
	
	PDB	X	X	http://www.pdb.org/
	
	IntAct	X	X	http://www.ebi.ac.uk/intact/

Homology	InterPro	X	X	http://www.ebi.ac.uk/interpro/
	
	PFam	X		http://pfam.sanger.ac.uk/
	
	PRINTS	X		http://www.bioinf.manchester.ac.uk/dbbrowser/PRINTS
	
	PROSITE	X	X	http://www.expasy.ch/prosite/
	
	PANTHER	X		http://www.pantherdb.org/
	
	ProDom	X		http://prodom.prabi.fr/

Pathway	Reactome	X		http://www.reactome.org/
	
	KEGG Pathway	X	X	http://www.kegg.jp/
	
	BioCyc	X	X	http://www.biocyc.org/

Drug	KEGG Drug	X	X	http://www.kegg.jp/
	
	PharmGKB	X	X	http://www.pharmgkb.org/
	
	DrugBank	X		http://www.drugbank.ca/

Disease	OMIM	X		http://www.ncbi.nlm.nih.gov/omim/
	
	Orphanet	X		http://www.orpha.net/

Enzyme	EC	X		http://www.biochem.ucl.ac.uk/bsm/dbbrowser/protocol/ecenzfrm.html
	
	Brenda	X		http://www.brenda-enzymes.org/

Bibliography	PubMed	X	X	http://www.ncbi.nlm.nih.gov/pubmed/

Ontology	Gene Ontology	X		http://www.geneontology.org/

### Background processing

As previously mentioned, one of the requirements in developing GeneBrowser was the need to offer a low response time to user requests. While the use of GeNS was a major step towards meeting this goal, some tools possess relatively heavy processing needs that require fine-tuning. This is the case of computing the GO directed acyclic graph (DAG) and the bibliographical list. Because two out of the seven functionalities provided are computationally intensive, and as such, cannot be made immediately available after submitting the dataset, their processing is executed in the background and it is made available as soon as it is complete.

After insertion of a new dataset, GeneBrowser launches a background process that pre-computes the *p-value *for each entry and stores it in the database. While all the other tools are made available immediately, ontology and bibliographical options may trigger a message informing that the values are still being processed. For registered users, future access to the dataset will not require reprocessing because the results are permanently stored.

### Extended approach to functional gene clustering

Gene Ontology is the most relevant biological ontology, containing structured information about biological processes, cellular components and molecular functions. It is commonly used by establishing a match between genes in the dataset and terms in the ontology. The terms that accumulate higher number of genes are the ones with more potential interest to the study. To be valid, this gene accumulation procedure requires the use of statistical measures that consider the number of expected genes in each category and the occurrence of several simultaneous tests [21-23]. Despite the major relevance of Gene Ontology, other terminologies can be used to extract communalities from a dataset. Herein, we extend the use of this approach to pathways, protein domains, orthologues and homologues.

The implemented procedure works as follows. For each term *t *of a specific terminology, we obtain the associated genes from list *L1 *(representing the genes of interest) and the genes from list *L2 *(containing all genes under study - by default, all the genes from the genome). Then, for each term, we use the number of associated genes to calculate the *p-value*. The *p-value *is a measure of confidence in the data that corresponds to the probability of observing the actual number of genes in a certain category just by chance. Lower *p-values *indicate more confidence in the data, and usually only terms with values under 0.05 are considered.

Although several methods are available to calculate the *p-value*, GeneBrowser utilises binomial distribution, mainly due to its good balance between performance and robustness. A discussion of the available methods for gene expression studies is available in [22]. Because the *p-values *for all categories are calculated separately, the final step consists of adjusting the *p-values *to consider the occurrence of multi-testing [24]. GeneBrowser uses the false discovery rate (FDR) correction proposed by Benjamini and Yekutieli [23].

## Results

This section contains detailed information about the main functionalities provided in GeneBrowser.

### Inserting and exploring the dataset

The first step in using GeneBrowser is creating a new dataset. The information includes the species name of the organism, the list of genes found as relevant and a second list with all the genes under study (Figure 2). The list of supported species was obtained from the KEGG Organism database [25], selecting only those with complete genome annotation, resulting in a total of 1200 entries. Although GeneBrowser is prepared to deal with identifiers from all organisms, some functionalities depend on availability of the data in the original source.

Currently, GeneBrowser is prepared to accept 27 different types of gene identifiers simultaneously. The process of validating each gene identifier requires not only the removal of repeated and null entries but also their conversion to a unique internal identifier. Use of the GeNS name-server capabilities ensures this task is quickly performed, thus meeting the strict time requirements. The second list is used to calculate the significance values obtained through the statistical analysis algorithms. By default, this list contains all the genes in the genome.

Although registration is not mandatory, by logging on, the user can store all of the inserted datasets, thus saving time in future access. It is also possible to insert the dataset, close the browser and return later when the results are ready.

**Figure 3:**
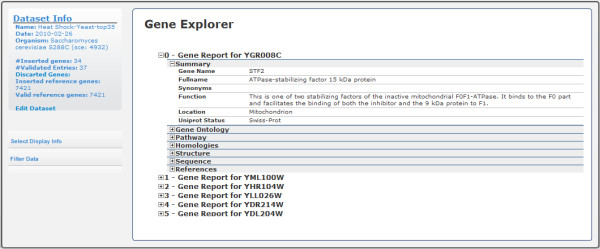
**GeneBrowser user-interface: gene explorer**. This interface contains the list of inserted genes, and for each, the most relevant biological data grouped in seven distinct sections: summary, gene ontology, pathway, homologies, protein structure, sequence, and external references. It is also possible to hide some sections and to filter the list of genes based on characteristics of the genes.

After inserting data, the Data Explorer facility provides a simple and effective way to visually inspect the inserted list of genes (Figure 3). For each gene, all of the information available on public databases is matched into seven distinct sections: general gene information, ontology classes, protein structure, genetic and protein sequence, bibliographical references and links to external databases.

Additionally, if the user is focused on a particular subject, it is possible to filter the initial list of genes for those that match the search criteria, such as a chromosome locus, Gene Ontology class or metabolic pathway.

### Gene clustering to extract similarities

The accumulation of genes into classes can be used to identify the most relevant biological phenomena. GeneBrowser uses a graphics-based view to allow immediate analysis of the most relevant functional classes. The significance is calculated using both the binomial and corrected *p-value*. Each class will show the number of accumulated genes, the *p-values*, and a direct link to the database that contains detailed information regarding that class. Next, we describe the available functionalities that use this clustering strategy: Gene Ontology, Homology, Pathway Explorer and Gene on Locus.

**Figure 4:**
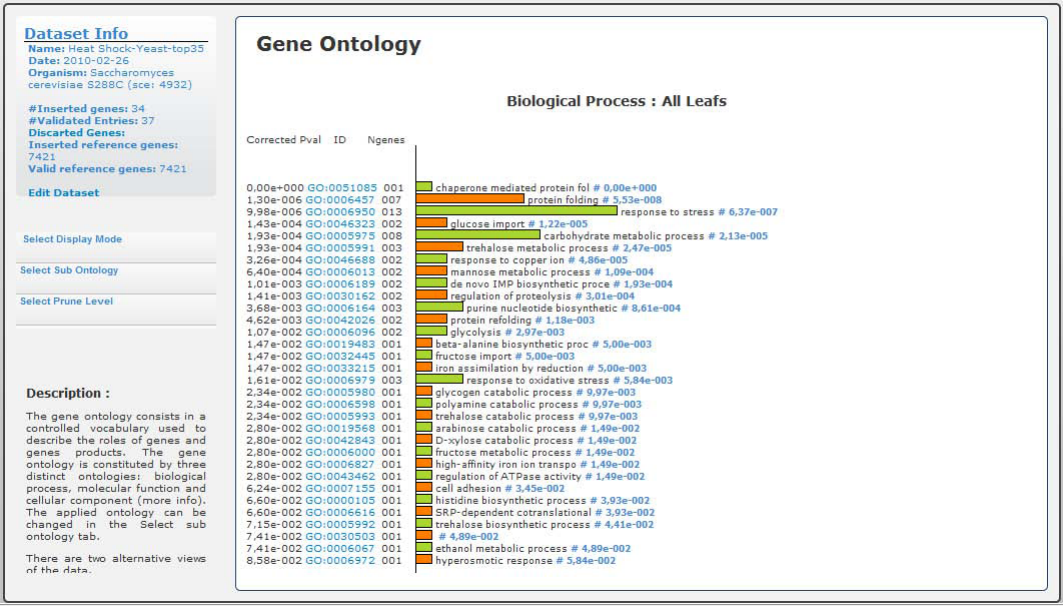
**GeneBrowser user-interface: gene ontology (graph view)**. This interface contains the graph with the list of Gene Ontology classes associated with the list of inserted genes. The graph displays the corrected *p-value*, the term identifiers with a link to the AmiGO database, the term name and the *p-value*.

**Figure 5:**
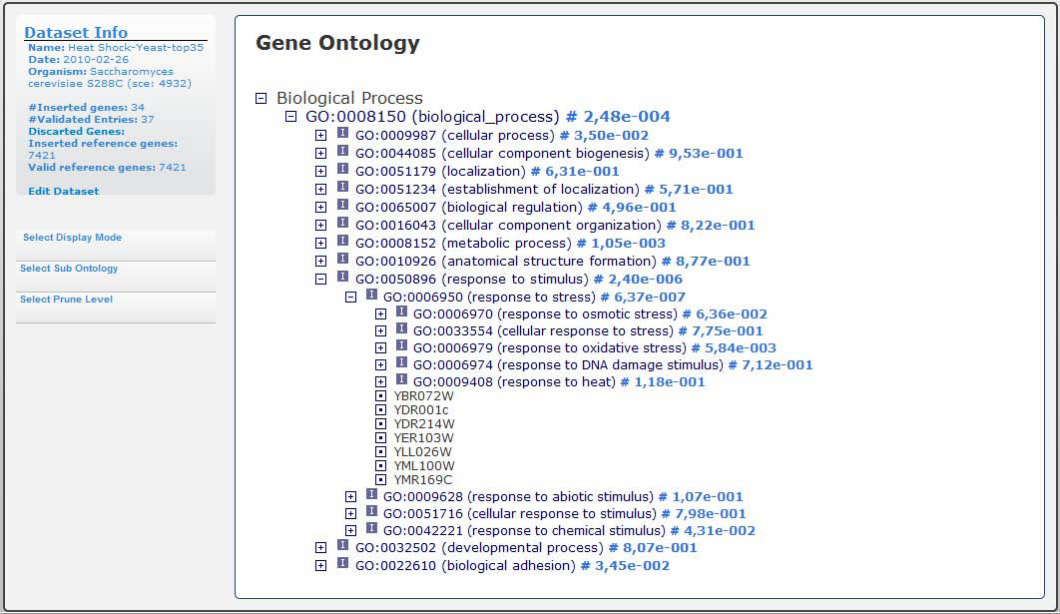
**GeneBrowser user-interface: gene ontology (tree view)**. This tree-based alternative is used to navigate Gene Ontology. Each branch contains the gene ontology identifier, the term name and the *p-value*.

Gene Ontology consists of a structured, controlled vocabulary used to describe genes and gene products independently of the organisms in which they are present. Because Gene Ontology is structured as a DAG, the graph view shows the most relevant terms independently of their relevance (Figure 4). For instance, due to lack of detail, a gene may be annotated as "Response to stress", while in fact, it is directly involved in a more specific stress such as "Response to heat". To address this issue, GeneBrowser proposes two solutions. First, GeneBrowser allows pruning of the DAG to a user-defined level where all pruned terms are enclosed in their more general counterparts. For instance, both "Response to heat" and "Response to oxidative stress" belong to level 3 in the ontology. By pruning the tree in level 2, genes previously annotated in those two classes are now considered in the more general term "Response to stress". Second, GeneBrowser allows navigation in the DAG by means of a tree that allows exploration of classes while keeping their structure in a familiar way (Figure 5).

Two genes are considered homologues if they share a common ancestor. Usually, homology is based on sequence similarity that can be inferred by computational methods (e.g.: BLAST, FASTA). These results can be stored in well-established databases that maintain the association between genes and their homologues. These repositories fall into two main categories: orthologues and protein subcomponents. The latter category includes motifs and domain (both structural and functional) databases [26]. For GeneBrowser, we selected six of the most relevant homology databases: PRINTS, PROSITE, KEGG Orthologs, ProDom, TIGRFAM and InterPro.

Analysing metabolic pathways allows biologists to know the processes in which genes are involved, much like Gene Ontology. Moreover, it also enables the creation of a graph of interactions in which the genes are involved. Pathway Explorer calculates the most relevant pathways and presents a graphical view of each pathway, highlighting the genes from the dataset.

Study of the distribution of genes is based on the assumption that co-expressed genes tend to be near one another in the genome. To validate this assumption, the identification of co-expressed genes in nearby loci is an important feature. For that, chromosomes are ordered by their significance showing the number of accumulated genes. From each chromosome, it is possible to navigate to the NCBI MapView tool and observe the genomic context of this particular set of genes. After this step, all the NCBI MapView features can be used to navigate in the chromosome.

### Extract relations from previous gene expression studies

The ArrayExpress Data Warehouse is the curated instance of the ArrayExpress repository [27]. By the end of 2009, it contained 1200 experiments with more than 31 million gene expression profiles. Because the data have been manually verified, they are of major relevance and can be used to study the expected profile of a set of genes. This database has already been used by the Gene Expression Atlas [28] to study the expression of several conditions such as different cell types, organism parts, developmental stages, disease states and sample treatments. Using the programmatic interface provided, we can establish a relationship between our list of genes and previous studies available in ArrayExpress.

**Figure 6:**
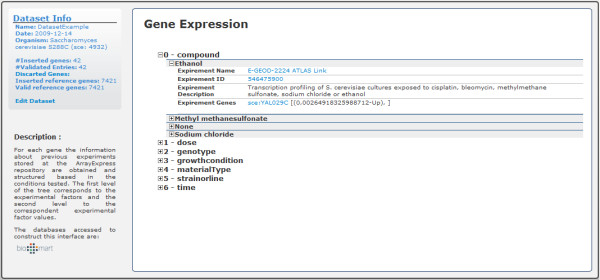
**GeneBrowser user-interface: gene expression**. This tree-based structure is composed of the experimental factor, associated factor values, and all genes in the context of the associated experiment.

The implemented procedure consists of obtaining, for each gene from the dataset, the experiments in which it was marked as differently expressed and the corresponding experimental conditions. Then, GeneBrowser organises the output in a structure composed of the experimental factor, the associated factor values, and all genes in the experiment (Figure 6). The interface also provides direct links to the experiment in ArrayExpress, to the study, and to the gene expression profile in Atlas.

### Literature mining

The MEDLINE database is the most relevant source of information for both biomedical and life sciences fields. Its broad collection included, at the end of 2009, more than 17 million abstracts collected from five thousand journals dating back to the 1960 s [29]. Every day, roughly 3000 new abstracts are added. To search and retrieve information from this database, an easy-to-use web interface named PubMed http://www.pubmed.com was developed. Herein, we use this huge array of data to obtain the most relevant papers for our dataset.

**Figure 7:**
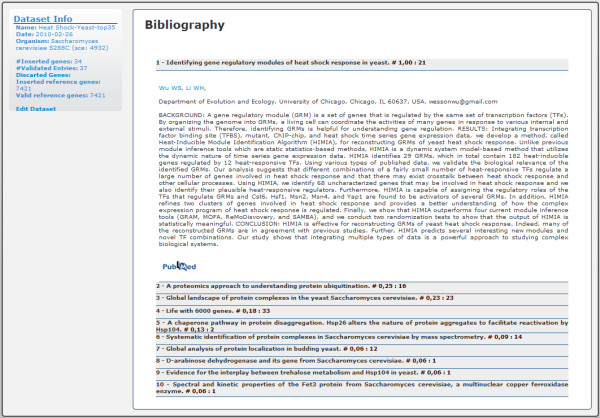
**GeneBrowser user-interface: literature**. This interface contains the ranked list of the papers in PubMed most closely related to the inserted dataset.

Ranking the papers by their relevance involves determining the relationship among three values: the total number of genes in the dataset, the total number of genes in each paper and the number of genes that are simultaneously in both the paper and the dataset. Ideally, a paper is relevant if the number of genes associated with it matches those from the dataset. To calculate the relevance, we used the Tanimoto coefficient, hence computing the similarity between the binary vectors formed by the genes present in each paper with the genes present in the dataset [30]. With representing the vector formed by the genes in the dataset and representing the vector with the genes in each paper, the relevance is given by:

The calculated values are subsequently normalised and their abstracts sorted and presented as illustrated in Figure 7.

## Discussion

The GeneBrowser tool can be used to analyse any set of genes. To show its added value, we used two sets obtained from expression experiments. The first one consisted of a heat shock experiment applied on yeast, and the second was a subset of the profiling of 47 human breast tumour cases.

### Heat shock on yeast

This experiment consisted of a comparison of two conditions, where one was the cell in a steady state and the other was after submitting it to a heat shock (delta of 20°C) for 20 minutes. For each condition, the total mRNA was extracted and three biological replicates were performed.

The expression values obtained for both conditions were analysed with a local framework [31] that uses the Limma library. Thirty-five genes were selected as differentially expressed and used to create a new dataset. This dataset is included as an example in GeneBrowser, and as such, the following steps can be followed directly through the application.

From the list of genes available in the Gene Explorer, it is easy to find the well-known gene YFL014W (also known as HSP12, *12-kDa heat shock protein*). Therefore, the user can easily obtain the list of bibliographical references, the genetic and genomic sequences, homologies and Gene Ontology terms related to this gene. It is also possible to verify the absence of data regarding pathways and protein structure. Using the provided functionalities, a possible step is to obtain all genes, including *YFL014W*, which are related to the response to oxidative stress. This task is performed using the filtering tools from the left menu and selecting the Gene Ontology term "*GO:0006979*". Other possible filtering options are pathways, homologies and chromosome.

An extended approach is available on the Gene Ontology tool. Figure 4 shows all biological processes with corrected *p-values *under 0.05. For this dataset, the two most relevant biological processes are the response to stress (with 9 genes) and protein folding (with 7 genes). After following the link to AmiGO, additional information can be observed, explaining that the response to stress is caused by an exogenous perturbation, being a direct consequence of the induced heat shock. Protein folding is a physical process in which a linear chain of polypeptides is folded to obtain the tertiary structure and to become a protein. Given that proteins unfold with increasing temperature, the observed induction is a feedback mechanism that helps to overcome this situation.

Additional information about the set of genes can also be found in the Bibliography section. For example, the top ranked paper "*Identifying gene regulatory modules of heat shock response in yeast*" relates directly to the subject of study (Figure 7). This experiment is publicly available in GeneBrowser through the URL http://bioinformatics.ua.pt/genebrowser2/Explorer.aspx?ID=441. The workflow of this study is provided in [Additional file 1].

### Experiment from ArrayExpress

The other experimental data were obtained from the ArrayExpress experiment "*Transcription profiling of 47 human breast tumor cases*", stored with the entry E-GEOD-3744 [32]. From this experiment, we selected all of the differently expressed genes associated with the experimental condition "BRCA1-*associated breast cancer*". The pathway analysis indicated that the most representative pathway was the "*Calcium signalling pathway*". In fact, several studies already stress the role of calcium in cancer as a major signalling agent, due to cell proliferation and cell death [33-35]. Another active pathway is the "*Pathways in cancer*" entry.

In the homology analysis, three protein families from the InterPro database were identified: rhodopsin-like superfamily, adrenergic receptor and nucleotide phosphodiesterase. Although the active genes were distributed across all chromosomes, the most active ones resided in chromosomes 1, 8 and 19. This experiment is also publicly available in GeneBrowser through the URL http://bioinformatics.ua.pt/genebrowser2/Explorer.aspx?ID=436. The workflow of this study is provided in [Additional file 2].

## Conclusions

A major challenge in bioinformatics is to construct tools that can give answers to any biological problem. One such problem is to interpret a list of genes, and find related diseases, pathways or any kind of biological process. This paper presents GeneBrowser, a web based application that, for a given list of genes, merges the benefits of biological web portals with those of knowledge extraction software tools. The main innovation of the proposed application is the possibility to use a single entry point to obtain a complete biological interpretation of the list of inserted genes, rather than using several disperse data sources and tools, thus facilitating biological interpretation of the study. The features of GeneBrowser include a gene explorer that enables simple and easy retrieval of specific molecular information for each inserted gene, thus allowing through filtering, the detection of common traits. The literature and the gene expression visualisation modes allow quick identification of previous related studies as well as an extended approach to functional analysis that facilitates detection of the most relevant classes in Gene Ontology, homologies, gene chromosomal localisation and pathways.

This second version includes a completely redesigned system and several improvements, including statistical analysis and multiple conceptually different kinds of gene identifiers such as input, chromosomal locus distribution, homologues and related gene expression studies. Possible future developments include the addition of regulatory information, miRNAs, and phenotype associations. Other feature that we aim to explore is to create a unified view that merges the different analysis outputs into a single one, providing a rich summary of the main evidences found by the several methods.

Overall, the functionalities implemented in GeneBrowser allow better understanding of the global biological phenomena underlying a list of genes.

## Availability and requirements

Project name: GeneBrowser

Project home page: http://bioinformatics.ua.pt/genebrowser2/

Operating system(s): Platform independent

Programming language: ASP.NET/C#

Other requirements: n/a

License: n/a Any restrictions to use by non-academics: no restrictions

## Authors' contributions

JA conceived and designed the application. JF wrote most of the code related to the interface, parallel processing and database. JP dealt with integrated access to external databases. JA, JP and JLO participated in writing the manuscript. All authors read and approved the final manuscript.

## Supplementary Material

Additional file 1 **Experiment "Heat shock on yeast"**. Workflow followed in GeneBrowser for the interpretation of the heat shock experiment. Click here for file

Additional file 2 **Experiment "*Transcription profiling of 47 human breast tumor cases*"**. Workflow followed in GeneBrowser for the interpretation of the breast tumor experiment. Click here for file
