# Supplementary material for: GeneBrowser 2: an application to explore and identify common biological traits in a set of genes
Source: BMC Bioinformatics. 2010 Jul 21;11:389. doi: 10.1186/1471-2105-11-389 (PMC2919517; doi:10.1186/1471-2105-11-389)
Supplement: Additional file 1 — Experiment "Heat shock on yeast". Workflow followed in GeneBrowser for the interpretation of the heat shock experiment. [file 1471-2105-11-389-S1.PDF]

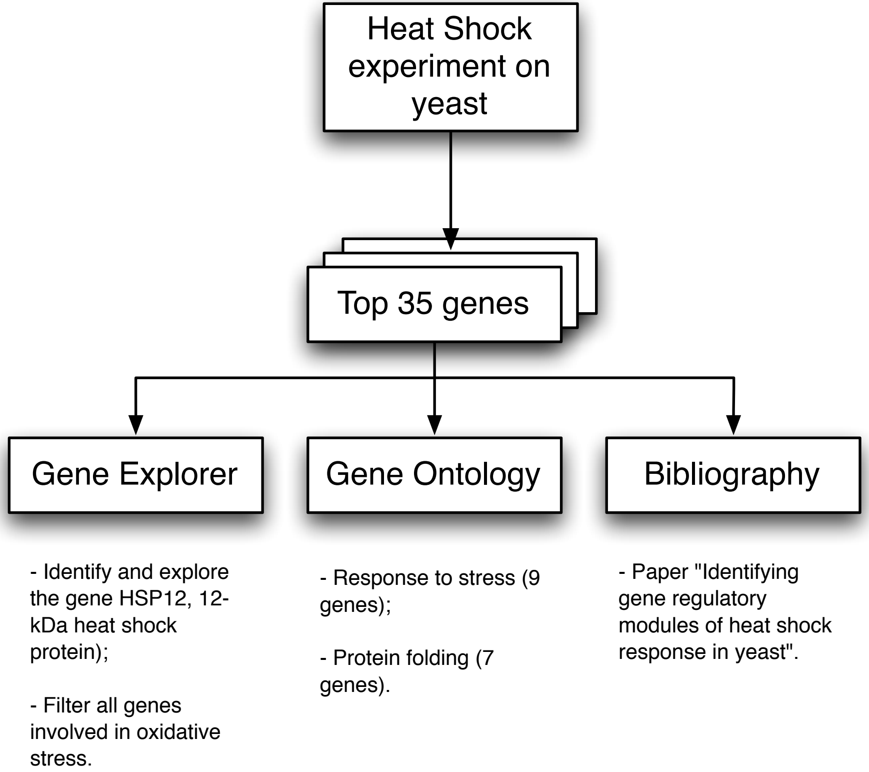
Heat Shock experiment on yeast
Top 35 genes
Gene Explorer
Gene Ontology
Bibliography
- Identify and explore the gene HSP12, 12-kDa heat shock protein);
- Filter all genes involved in oxidative stress.
- Response to stress (9 genes);
- Protein folding (7 genes).
- Paper "Identifying gene regulatory modules of heat shock response in yeast".
