# Supplementary material for: GeneBrowser 2: an application to explore and identify common biological traits in a set of genes
Source: BMC Bioinformatics. 2010 Jul 21;11:389. doi: 10.1186/1471-2105-11-389 (PMC2919517; doi:10.1186/1471-2105-11-389)
Supplement: Additional file 2 — Experiment "Transcription profiling of 47 human breast tumor cases". Workflow followed in GeneBrowser for the interpretation of the breast tumor experiment. [file 1471-2105-11-389-S2.PDF]

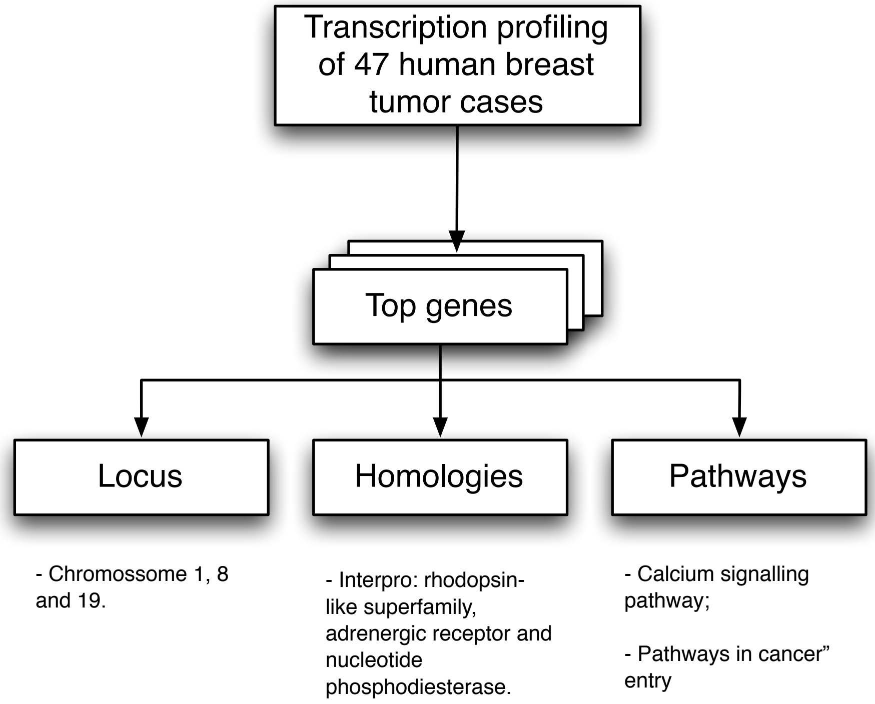
Transcription profiling of 47 human breast tumor cases
Top genes
Locus
Homologies
Pathways
- Chromossome 1, 8 and 19.
- Interpro: rhodopsin-like superfamily, adrenergic receptor and nucleotide phosphodiesterase.
- Calcium signalling pathway;
- Pathways in cancer" entry
